# Supplementary material for: Integration of Phosphoproteomics and Transcriptome Studies Reveals ABA Signaling Pathways Regulate UV-B Tolerance in Rhododendron chrysanthum Leaves
Source: Genes (Basel). 2023 May 25;14(6):1153. doi: 10.3390/genes14061153 (PMC10297884; doi:10.3390/genes14061153)
Supplement: Supplementary file 1 [file genes-14-01153-s001.zip › genes-2410791-supplementary.pdf]

Table S1. Differential phosphorylated protein table of *R. chrysanthum* under UV-B stress

| Protein accession | Position | Amino acid | Protein description | KEGG Gene | P value | Regulated Type |
|---|---|---|---|---|---|---|
| Gene.11338_CL835.Contig6_All | 184 | S | RPM1-interacting protein 4 | RIN4; RPM1-interacting protein 4 | 0.0069796 | Down |
| Gene.11338_CL835.Contig6_All | 86 | S | RPM1-interacting protein 4 | RIN4; RPM1-interacting protein 4 | 0.010177 | Down |
| Gene.13985_CL1105.Contig1_All | 109 | S | "Plastid-lipid-associated protein 6, chloroplastic" | | 0.0023211 | Down |
| Gene.13985_CL1105.Contig1_All | 220 | S | "Plastid-lipid-associated protein 6, chloroplastic" | | 0.041461 | Up |
| Gene.14291_CL1144.Contig3_All | 921 | T | PREDICTED: uncharacterized protein LOC100250143 isoform X1 [Vitis vinifera] | | 0.028078 | Down |
| Gene.14602_CL1180.Contig5_All | 11 | S | 60S ribosomal protein L6 | RP-L6e; large subunit ribosomal protein L6e | 0.00081575 | Down |
| Gene.14686_CL1191.Contig2_All | 320 | S | ABC transporter C family member 5 | | 0.032404 | Up |
| Gene.15319_CL1247.Contig1_All | 107 | T | "Transketolase, chloroplastic" | E2.2.1.1; transketolase [EC:2.2.1.1] | 0.0023804 | Up |
| Gene.19346_CL1703.Contig8_All | 154 | S | Serine/threonine-protein kinase SAPK2 | SNRK2; serine/threonine-protein kinase SRK2 [EC:2.7.11.1] | 0.04842 | Up |
| Gene.20539_CL1824.Contig2_All | 135 | S | "Sec-independent protein translocase protein TATA, chloroplastic" | tatA; sec-independent protein translocase protein TatA | 0.035424 | Up |
| Gene.21159_CL1903.Contig2_All | 178 | S | "ATP synthase subunit b', chloroplastic" | ATPF0B; F-type H+-transporting ATPase subunit b | 0.000062784 | Up |

| | | | | | | |
|---|---|---|---|---|---|---|
| Gene.23080_CL2152.Contig1_All | 356 | T | Protein BTR1 | | 0.0059976 | Down |
| Gene.23829_CL2226.Contig3_All | 60 | S | CSC1-like protein HYP1 | | 0.035557 | Down |
| Gene.24603_CL2333.Contig3_All | 725 | S | Nuclear pore complex protein NUP98A | NUP98; nuclear pore complex protein Nup98-Nup96 | 0.023098 | Down |
| Gene.26206_CL2559.Contig3_All | 522 | S | Serine/threonine-protein kinase BLUS1 | | 0.029463 | Up |
| Gene.26206_CL2559.Contig3_All | 491 | S | Serine/threonine-protein kinase BLUS1 | | 0.0137422 | Up |
| Gene.26950_CL2640.Contig3_All | 398 | S | "Monodehydroascorbate reductase, chloroplastic/mitochondrial" | E1.6.5.4; monodehydroascorbate reductase (NADH) [EC:1.6.5.4] | 0.031282 | Down |
| Gene.27331_CL2704.Contig2_All | 77 | S | Chitin-binding lectin 1 | EIF2B4; translation initiation factor eIF-2B subunit delta | 0.044879 | Down |
| Gene.28080_CL2824.Contig6_All | 137 | S | PREDICTED: uncharacterized protein LOC105120307 isoform X2 [Populus euphratica] | | 0.04908 | Down |
| Gene.28776_CL2933.Contig2_All | 91 | T | Nucleoside diphosphate kinase 1 | ndk; nucleoside-diphosphate kinase [EC:2.7.4.6] | 0.0022381 | Up |
| Gene.29020_CL2993.Contig9_All | 100 | S | Isocitrate dehydrogenase [NADP] | IDH1; isocitrate dehydrogenase [EC:1.1.1.42] | 0.0037984 | Down |

| | | | | | | |
|---|---|---|---|---|---|---|
| Gene.30247_CL3160.Contig4_All | 3 | S | Nitrate regulatory gene2 protein | | 0.021555 | Down |
| Gene.31339_CL3346.Contig6_All | 369 | T | Monosaccharide-sensing protein 2 | | 0.0044396 | Up |
| Gene.31339_CL3346.Contig6_All | 419 | S | Monosaccharide-sensing protein 2 | | 0.0120847 | Up |
| Gene.32668_CL3593.Contig4_All | 107 | T | "PGR5-like protein 1B, chloroplastic" | | 0.0008845 | Down |
| Gene.33054_CL3661.Contig1_All | 422 | T | ABSCISIC ACID-INSENSITIVE 5-like protein 5 | ABF; ABA responsive element binding factor | 0.0020837 | Up |
| Gene.34110_CL3830.Contig1_All | 250 | S | MAP3K epsilon protein kinase 1 | | 0.0040816 | Up |
| Gene.34124_CL3832.Contig4_All | 95 | T | "RuBisCO large subunit-binding protein subunit alpha, chloroplastic" | | 0.0046957 | Up |
| Gene.34496_CL3892.Contig8_All | 240 | S | Auxin efflux carrier component 7 | | 0.0054587 | Up |
| Gene.34715_CL3946.Contig2_All | 387 | S | 60S acidic ribosomal protein P0 | RP-LP0; large subunit ribosomal protein LP0 | 0.022636 | Down |
| Gene.3570_CL223.Contig5_All | 10 | S | PREDICTED: conserved oligomeric Golgi complex subunit 1 [Vitis vinifera] | | 0.00076117 | Down |
| Gene.36597_CL4335.Contig4_All | 88 | S | "Phosphoglycerate kinase, chloroplastic" | PGK; phosphoglycerate kinase [EC:2.7.2.3] | 0.000083525 | Up |
| Gene.36597_CL4335.Contig4_All | 86 | T | "Phosphoglycerate kinase, chloroplastic" | PGK; phosphoglycerate kinase [EC:2.7.2.3] | 0.0032231 | Up |
| Gene.36604_CL4338.Contig2_All | 409 | S | Protein SRC2 | | 0.0062008 | Up |
| Gene.37414_CL4489.Contig4_All | 77 | S | "Ferritin-1, chloroplastic" | | 0.0096954 | Up |
| Gene.38423_CL4705.Contig5_All | 214 | S | "Endo-1,3;1,4-beta-D-glucanase" | | 0.003559 | Up |

| | | | | | | |
|---|---|---|---|---|---|---|
| Gene.38510_CL4726.Contig1_All | 93 | S | Tobamovirus multiplication protein 2A | | 0.0040639 | Down |
| Gene.3887_CL243.Contig4_All | 439 | S | "Glyceraldehyde-3-phosphate dehydrogenase B, chloroplastic" | GAPA; glyceraldehyde-3-phosphate dehydrogenase (NADP+) (phosphorylating) [EC:1.2.1.13] | 0.0058047 | Up |
| Gene.3887_CL243.Contig4_All | 144 | S | "Glyceraldehyde-3-phosphate dehydrogenase B, chloroplastic" | GAPA; glyceraldehyde-3-phosphate dehydrogenase (NADP+) (phosphorylating) [EC:1.2.1.13] | 0.00195795 | Up |
| Gene.38972_CL4827.Contig1_All | 60 | S | "Fructose-bisphosphate aldolase 2, chloroplastic" | ALDO; fructose-bisphosphate aldolase, class I [EC:4.1.2.13] | 0.00032014 | Down |
| Gene.38985_CL4829.Contig2_All | 221 | S | "Gamma carbonic anhydrase 1, mitochondrial" | | 0.025757 | Down |
| Gene.39709_CL5012.Contig3_All | 41 | S | Polyadenylation and cleavage factor homolog 1 | PCF11; pre-mRNA cleavage complex 2 protein Pcf11 | 0.004624 | Down |
| Gene.40269_CL5129.Contig3_All | 181 | T | "20 kDa chaperonin, chloroplastic" | | 0.010884 | Up |
| Gene.40497_CL5173.Contig2_All | 78 | S | Phosphoprotein ECPP44 | | 0.04812 | Down |
| Gene.4136_CL252.Contig3_All | 649 | S | "Glucose-6-phosphate isomerase 1, chloroplastic" | GPI; glucose-6-phosphate isomerase [EC:5.3.1.9] | 0.0159407 | Down |
| Gene.42653_CL5697.Contig2_All | 1408 | S | Protein transport protein SEC16B homolog | | 0.0109043 | Down |

| | | | | | | |
|---|---|---|---|---|---|---|
| Gene.43342_CL5854.Contig2_All | 146 | S | "Peroxiredoxin Q, chloroplastic" | | 0.00033787 | Up |
| Gene.43600_CL5931.Contig3_All | 36 | S | MLP-like protein 328 | | 0.000058716 | Down |
| Gene.44435_CL6125.Contig3_All | 47 | T | "Phosphoribulokinase, chloroplastic" | PRK; phosphoribulokinase [EC:2.7.1.19] | 0.003202 | Down |
| Gene.45512_CL6407.Contig3_All | 519 | T | Leucine-rich repeat extensin-like protein 3 | ACIN1; apoptotic chromatin condensation inducer in the nucleus | 0.0029379 | Up |
| Gene.45512_CL6407.Contig3_All | 556 | S | Leucine-rich repeat extensin-like protein 3 | ACIN1; apoptotic chromatin condensation inducer in the nucleus | 0.000037221 | Down |
| Gene.45512_CL6407.Contig3_All | 526 | S | Leucine-rich repeat extensin-like protein 3 | ACIN1; apoptotic chromatin condensation inducer in the nucleus | 0.000035816 | Up |
| Gene.46142_CL6569.Contig6_All | 165 | S | "1,4-alpha-glucan-branching enzyme 2-2, chloroplastic/amyloplastic" | GBE1; 1,4-alpha-glucan branching enzyme [EC:2.4.1.18] | 0.00044421 | Down |
| Gene.46959_CL6776.Contig1_All | 168 | T | RPM1-interacting protein 4 | RIN4; RPM1-interacting protein 4 | 0.0174989 | Up |
| Gene.46959_CL6776.Contig1_All | 163 | T | RPM1-interacting protein 4 | RIN4; RPM1-interacting protein 4 | 0.04094 | Up |
| Gene.47171_CL6821.Contig2_All | 265 | S | hypothetical protein Ccrd_008086 [Cynara cardunculus var. scolymus] | BABAM; BRISC and BRCA1-A complex member 1 | 0.0025644 | Down |

| | | | | | | |
|---|---|---|---|---|---|---|
| Gene.47456_CL6876.Contig2_All | 454 | S | Protein phosphatase 2C and cyclic nucleotide-binding/kinase domain-containing protein | | 0.011857 | Down |
| Gene.48804_CL7147.Contig2_All | 328 | S | Eukaryotic translation initiation factor 4B1 | | 0.000020188 | Down |
| Gene.49846_CL7399.Contig2_All | 58 | S | SUN domain-containing protein 1 | | 0.018705 | Down |
| Gene.49846_CL7399.Contig2_All | 48 | S | SUN domain-containing protein 1 | | 0.021356 | Down |
| Gene.51486_CL7752.Contig8_All | 64 | S | "Photosystem II 10 kDa polypeptide, chloroplastic" | psbR; photosystem II 10kDa protein | 0.0044599 | Down |
| Gene.52037_CL7839.Contig2_All | 26 | S | Annexin D4 | | 0.0082023 | Down |
| Gene.53321_CL8052.Contig1_All | 271 | T | Heat shock cognate 70 kDa protein 2 | HSPA1s; heat shock 70kDa protein 1/2/6/8 | 0.00187768 | Down |
| Gene.55228_CL8212.Contig5_All | 3 | S | V-type proton ATPase catalytic subunit A | ATPeV1A; V-type H+-transporting ATPase subunit A [EC:7.1.2.2] | 0.0052807 | Down |
| Gene.56696_CL8337.Contig10_All | 392 | S | "Chaperonin 60 subunit beta 2, chloroplastic" | groEL; chaperonin GroEL | 0.031479 | Up |
| Gene.57775_Unigene284_All | 96 | S | Probable calcium-binding protein CML18 | CALM; calmodulin | 0.039236 | Down |
| Gene.58581_Unigene831_All | 67 | S | Metacaspase-4 | | 0.00036166 | Down |
| Gene.58589_Unigene835_All | 783 | S | EH domain-containing protein 2 | | 0.027299 | Down |
| Gene.64089_Unigene5111_All | 236 | S | Uncharacterized protein At1g03900 | | 0.0032601 | Down |
| Gene.65018_Unigene5787_All | 524 | S | GBF-interacting protein 1 | | 0.0108382 | Up |

| | | | | | | |
|---|---|---|---|---|---|---|
| Gene.65697_Unigene6211_All | 232 | S | Photosystem II protein D1 | psbA; photosystem II P680 reaction center D1 protein [EC:1.10.3.9] | 0.000024507 | Up |
| Gene.66649_Unigene6892_All | 237 | S | "RNA pseudouridine synthase 6, chloroplastic" | | 0.0090224 | Down |
| Gene.68254_Unigene8007_All | 178 | S | Protein BONZAI 1 | | 0.00063999 | Down |
| Gene.6848_CL464.Contig2_All | 6 | S | Probable aquaporin PIP2-8 | | 0.00119818 | Down |
| Gene.6848_CL464.Contig2_All | 282 | T | Probable aquaporin PIP2-8 | | 0.0116366 | Down |
| Gene.69502_Unigene8888_All | 119 | S | 187-kDa microtubule-associated protein AIR9 | | 0.032743 | Down |
| Gene.70938_Unigene9872_All | 291 | S | "Membrane-associated 30 kDa protein, chloroplastic" | | 0.0041569 | Down |
| Gene.71075_Unigene9967_All | 51 | S | "Linoleate 13S-lipoxygenase 2-1, chloroplastic" | LOX2S; lipoxygenase [EC:1.13.11.12] | 0.000078486 | Down |
| Gene.71128_Unigene9997_All | 206 | S | Acyl-CoA-binding domain-containing protein 1 | | 0.0051022 | Down |
| Gene.71430_Unigene10207_All | 534 | S | Dynamin-2B | DNM; dynamin GTPase [EC:3.6.5.5] | 0.000098455 | Down |
| Gene.71430_Unigene10207_All | 881 | S | Dynamin-2B | DNM; dynamin GTPase [EC:3.6.5.5] | 0.012035 | Down |
| Gene.71879_Unigene10600_All | 190 | S | uncharacterized protein LOC107468142 [Arachis duranensis] | | 0.028077 | Up |

| | | | | | | |
|---|---|---|---|---|---|---|
| Gene.72388_Unigene11314_All | 155 | S | hypothetical protein [Camellia sinensis] | | 0.00108409 | Down |
| Gene.72474_Unigene11360_All | 95 | S | Mitochondrial outer membrane protein porin of 34 kDa | | 0.00113557 | Down |
| Gene.72804_Unigene11573_All | 131 | S | hypothetical protein VITISV_016468 [Vitis vinifera] | | 0.0049434 | Down |
| Gene.73161_Unigene11817_All | 157 | S | Methyl-CpG-binding domain-containing protein 13 | | 0.0075173 | Down |
| Gene.73161_Unigene11817_All | 167 | S | Methyl-CpG-binding domain-containing protein 13 | | 0.039717 | Down |
| Gene.73460_Unigene12021_All | 60 | S | "Peroxiredoxin-2F, mitochondrial" | | 0.047801 | Up |
| Gene.73584_Unigene12104_All | 149 | T | Exocyst complex component EXO70B2 | | 0.0038577 | Up |
| Gene.73621_Unigene12126_All | 266 | S | "Oxygen-dependent coproporphyrinogen-III oxidase, chloroplastic" | CPOX; coproporphyrinogen III oxidase [EC:1.3.3.3] | 0.0070391 | Up |
| Gene.73685_Unigene12173_All | 18 | S | Stem-specific protein TSJT1 | | 0.00062101 | Up |
| Gene.75286_Unigene13818_All | 171 | S | "50S ribosomal protein L29, chloroplastic" | RP-L29; large subunit ribosomal protein L29 | 0.00170379 | Down |
| Gene.75292_Unigene13824_All | 56 | S | Reticulon-like protein B4 | | 0.035724 | Up |
| Gene.7572_CL536.Contig1_All | 44 | S | 18.2 kDa class I heat shock protein | HSP20; HSP20 family protein | 0.0051778 | Down |
| Gene.7619_CL543.Contig2_All | 217 | S | Glycine-rich protein 2 | | 0.00160312 | Down |

| | | | | | | |
|---|---|---|---|---|---|---|
| Gene.76858_Unigene15319_All | 164 | S | Probable ADP-ribosylation factor GTPase-activating protein AGD6 | ARFGAP1; ADP-ribosylation factor GTPase-activating protein 1 | 0.011138 | Down |
| Gene.77037_Unigene15420_All | 466 | S | PREDICTED: putative G3BP-like protein isoform X1 [Nicotiana attenuata] | | 0.022538 | Down |
| Gene.77355_Unigene15616_All | 240 | S | 40S ribosomal protein S6 | RP-S6e; small subunit ribosomal protein S6e | 0.00084111 | Up |
| Gene.77538_Unigene15752_All | 257 | S | Pyruvate decarboxylase 1 | PDC; pyruvate decarboxylase [EC:4.1.1.1] | 0.000163649 | Down |
| Gene.79431_Unigene17536_All | 43 | S | protein SSUH2 homolog [Manihot esculenta] | | 0.0151808 | Up |
| Gene.7986_CL578.Contig2_All | 81 | S | "Photosystem II 10 kDa polypeptide, chloroplastic" | psbR; photosystem II 10kDa protein | 1.76886E-05 | Down |
| Gene.7986_CL578.Contig2_All | 73 | S | "Photosystem II 10 kDa polypeptide, chloroplastic" | psbR; photosystem II 10kDa protein | 0.0049196 | Down |
| Gene.80537_Unigene18753_All | 549 | S | Flowering time control protein FPA | | 0.033263 | Down |
| Gene.80865_Unigene18952_All | 519 | T | Kinesin-like protein KIN-14B | | 0.000060418 | Up |
| Gene.80865_Unigene18952_All | 660 | S | Kinesin-like protein KIN-14B | | 0.010517 | Down |
| Gene.81168_Unigene19137_All | 29 | S | PREDICTED: uncharacterized protein LOC102598108 [Solanum tuberosum] | HSPBP1; hsp70-interacting protein | 0.0022984 | Down |

| | | | | | | |
|---|---|---|---|---|---|---|
| Gene.81465_Unigene19343_All | 330 | S | Proline-rich receptor-like protein kinase PERK2 | | 0.0091392 | Down |
| Gene.81465_Unigene19343_All | 105 | T | Proline-rich receptor-like protein kinase PERK2 | | 0.002197 | Down |
| Gene.81522_Unigene19369_All | 80 | T | Leucine-rich repeat extensin-like protein 3 | | 0.00034394 | Down |
| Gene.81531_Unigene19376_All | 621 | S | Eukaryotic translation initiation factor isoform 4G-2 | EIF4G; translation initiation factor 4G | 0.00044077 | Down |
| Gene.81531_Unigene19376_All | 61 | S | Eukaryotic translation initiation factor isoform 4G-2 | EIF4G; translation initiation factor 4G | 0.000035394 | Down |
| Gene.81531_Unigene19376_All | 184 | S | Eukaryotic translation initiation factor isoform 4G-2 | EIF4G; translation initiation factor 4G | 0.00133917 | Down |
| Gene.81544_Unigene19383_All | 55 | S | "Fructose-bisphosphate aldolase 6, cytosolic" | ALDO; fructose-bisphosphate aldolase, class I [EC:4.1.2.13] | 0.0084593 | Up |
| Gene.82839_Unigene20755_All | 608 | S | Heat shock 70 kDa protein 16 | | 0.00027969 | Down |
| Gene.83286_Unigene21046_All | 51 | T | hypothetical protein TSUD_229720 [Trifolium subterraneum] | | 0.00081924 | Down |
| Gene.83366_Unigene21103_All | 951 | S | "Histidine--tRNA ligase, cytoplasmic" | HARS; histidyl-tRNA synthetase [EC:6.1.1.21] | 0.0028558 | Down |
| Gene.85388_Unigene22921_All | 159 | S | 2-methylene-furan-3-one reductase | | 1.68112E-05 | Up |
| Gene.85395_Unigene22926_All | 63 | S | "Protein CURVATURE THYLAKOID 1A, chloroplastic" | | 0.0050235 | Down |

| | | | | | | |
|---|---|---|---|---|---|---|
| Gene.85450_Unigene22951_All | 33 | S | Probable serine/threonine-protein kinase SIS8 | | 0.027439 | Down |
| Gene.85450_Unigene22951_All | 1159 | T | Probable serine/threonine-protein kinase SIS8 | | 0.0020803 | Down |
| Gene.85630_Unigene23100_All | 152 | S | Cold shock protein 2 | | 0.0051199 | Down |
| Gene.86177_Unigene24148_All | 140 | S | Formin-like protein 14 | | 0.0148779 | Down |
| Gene.87528_Unigene32298_All | 37 | T | "Chlorophyll a-b binding protein CP26, chloroplastic" | LHCB5; light-harvesting complex II chlorophyll a/b binding protein 5 | 0.00043511 | Down |
| Gene.8795_CL667.Contig11_All | 203 | T | Protein NTM1-like 9 | | 0.032002 | Down |
| Gene.8795_CL667.Contig11_All | 510 | T | Protein NTM1-like 9 | | 0.038898 | Down |
| Gene.8795_CL667.Contig11_All | 14 | S | Protein NTM1-like 9 | | 0.024445 | Down |
| Gene.9131_CL681.Contig8_All | 68 | S | Uncharacterized membrane protein At1g16860 | | 0.0103017 | Down |
| Gene.9131_CL681.Contig8_All | 72 | S | Uncharacterized membrane protein At1g16860 | KEGG Gene | 0.00150186 | Down |
